# Periodontitis-associated septic pulmonary embolism caused by *Actinomyces species* identified by anaerobic culture of bronchoalveolar lavage fluid: a case report

**DOI:** 10.1186/s12879-015-1286-0

**Published:** 2015-12-01

**Authors:** Shun Endo, Eikan Mishima, Yoichi Takeuchi, Takashi Ohi, Masatsugu Ishida, Masaru Yanai, Hideyasu Kiyomoto, Tasuku Nagasawa, Sadayoshi Ito

**Affiliations:** Department of Internal Medicine, Japanese Ishinomaki Red Cross Hospital, Ishinomaki, Japan; Department of Dentistry, Japanese Ishinomaki Red Cross Hospital, Ishinomaki, Japan; Department of Respiratory Medicine, Japanese Ishinomaki Red Cross Hospital, Ishinomaki, Japan; Tohoku Medical Megabank Organization, Tohoku University, Sendai, Japan; Division of Nephrology, Endocrinology, and Vascular Medicine, Tohoku University Graduate School of Medicine, Sendai, Japan; Division of Aging and Geriatric Dentistry, Department of Oral Function and Morphology, Tohoku University Graduate School of Dentistry, Sendai, Japan

**Keywords:** Septic pulmonary embolism, Periodontitis, *Actinomyces species*, Dental infection, Bronchoalveolar lavage, Anaerobic culture, Chest pain

## Abstract

**Background:**

Periodontal disease is a less common but important cause of septic pulmonary embolism (SPE). However, the pathogens causing periodontal disease-associated SPE (PD-SPE) have been poorly understood. *Actinomyces species* are resident microbiota in the oral cavity. Here we report a case of PD-SPE caused by *Actinomyces species*, which was identified by anaerobic culture of bronchoalveolar lavage fluid (BAL).

**Case presentation:**

A 64-year-old Asian man, complicated with severe chronic periodontitis, was admitted with chest pain and fever. Chest CT revealed multiple bilateral pulmonary nodules located subpleurally. We diagnosed the case as SPE associated with periodontitis. Although blood cultures were negative for the usual 5-day incubation, anaerobic culture of the BAL fluid sample yielded *Actinomyces species.* Antibacterial therapy alone did not ameliorate the symptoms; however, additional dental treatment, including tooth extraction, promptly did. The patient was discharged 23 days after admission. The 3-month follow-up revealed no recurrence of the symptoms and complete resolution of the lung lesions.

**Conclusion:**

This case demonstrated that *Actinomyces species* can cause PD-SPE. Additionally, clinicians should consider performing appropriate anaerobic culture of BAL fluid to identify the pathogen of SPE, and to ordering dental treatment, if necessary, in addition to antibiotics for the initial management of PD-SPE.

## Background

Septic pulmonary embolism (SPE) is a serious disorder in which thrombi containing microorganisms in a fibrin matrix are mobilized from an infectious nidus and transported through the venous system to become implanted in the vascular system of the lungs [1]. SPE is usually associated with tricuspid valve endocarditis, infected central venous catheters, septic thrombophlebitis including Lemierre’s syndrome, and oropharyngeal infection [1]. Additionally, periodontal diseases such as periodontitis have been reported to be a less common but important cause of SPE [2, 3]. The causative pathogens of SPE vary depending on the infectious source [1]. However, the causative pathogens of the periodontal disease-associated SPE are poorly understood, likely because of inappropriate culturing and sample collection techniques [3, 4]. Here we report a case of periodontitis-associated SPE caused by *Actinomyces species*, which was identified by anaerobic culture of bronchoalveolar lavage (BAL) fluid. In the present case, tooth extraction was required for the initial treatment in addition to antimicrobial therapy.

## Case presentation

**Fig. 1:**
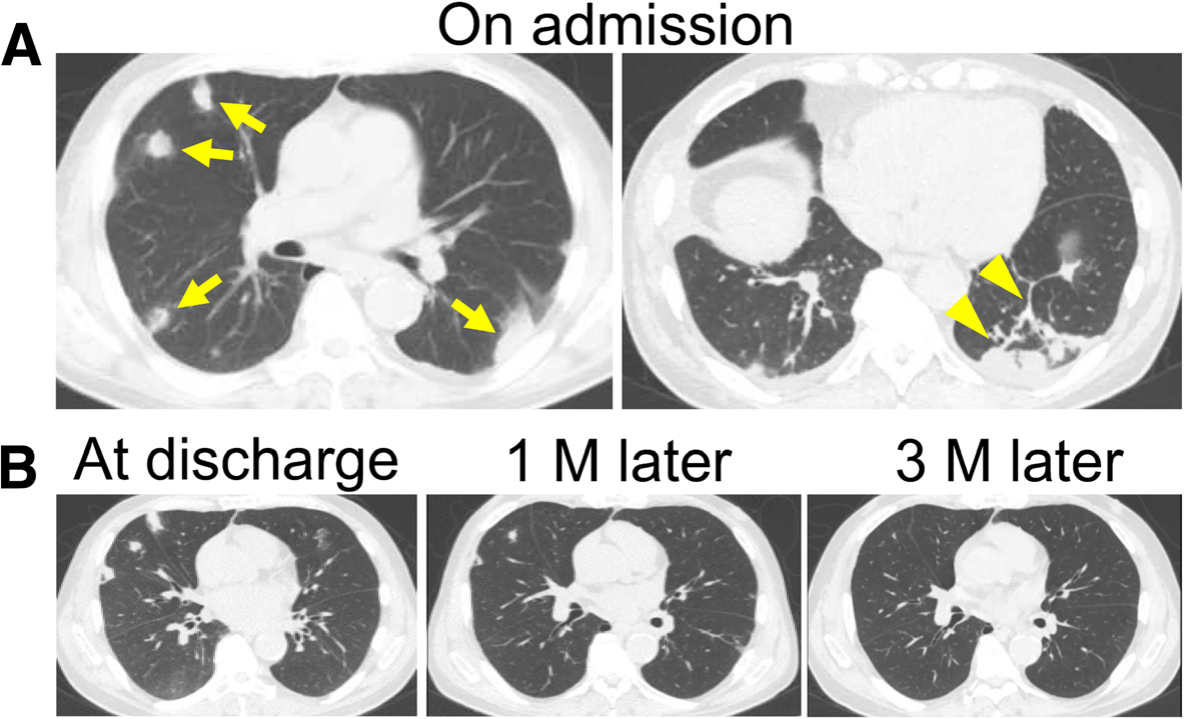
CT images of septic pulmonary emboli. **a** Chest CT images on admission. Arrows indicate septic embolic lesions. Arrowheads indicate a feeding vessel sign and a wedge-shaped peripheral lesion. **b** Follow-up images at discharge, 1 month and 3 months after the discharge

**Fig. 2:**
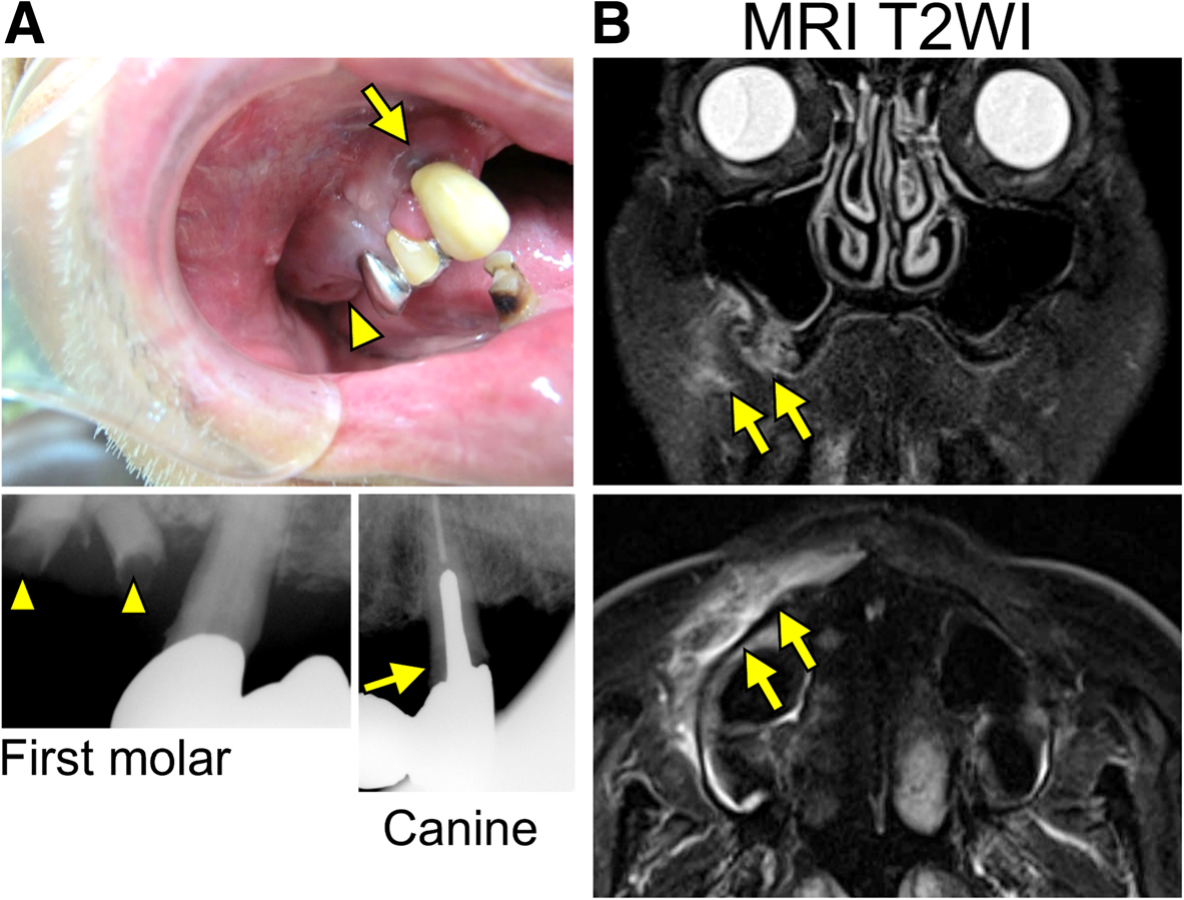
Images of periodontic lesions. **a** Intraoral and radiographic view of the right maxilla. Canine (*arrows*) and remaining roots of the first molar (*arrowheads*) are extracted as the subsequent treatment. **b** T2-weighted MR images of the face. Arrows indicate hyperintensities in the right maxilla

A 64-year-old man was brought to our hospital with a two-day history of worsening chest pain on inspiration, dyspnea and fever. The patient had a medical history of poorly-controlled diabetes mellitus (Hemoglobin A1c level, 9 %), and a history of heavy alcohol consumption but no past history of pulmonary disease. On admission, his vital signs were: blood pressure 138/98 mmHg, pulse rate 103 beats/min, body temperature 38.8 °C, respiratory rate 22 breaths/min, and arterial oxygen saturation 94 % on 2 L/min oxygen by nasal cannula. Laboratory examination showed leukocytosis (15,000 cells/μl) and elevated levels of C-reactive protein (24.2 mg/dL), fibrinogen (882 mg/dL) and D-dimer (3.86 μg/dL). A chest radiograph showed multiple, small pulmonary nodules in both lungs. Chest computed tomography (CT) revealed multiple bilateral pulmonary nodules, mostly located subpleurally (Fig. 1a). Some of these lesions showed feeding vessel signs and there were wedge-shaped peripheral lesions abutting the pleura (Fig. 1a), suggesting SPE [1]. In the search for the primary source of infection, transthoracic echocardiography showed no vegetations on the heart valves, and neck echography was normal. However, the patient complained of pain of the right upper jaw. Oral examination by a dentist revealed poor oral hygiene and severe chronic periodontitis, especially the right maxillary canine had remarkable gingival swelling, suppuration, ≥ 8 mm of probing pocket depth and radiographically confirmed severe bone loss that was horizontally widespread (Fig. 2a). Facial MRI furthermore revealed hyperintensity on T2-weighted image in the right alveopalatal part and maxilla (Fig. 2b), the same locations as the periodontitis, indicating inflammatory alterations. Therefore, we diagnosed the case as periodontitis-associated SPE and started empirical treatment with meropenem on hospital day 1. However, since the patient remained febrile (38 °C) and showed deteriorated chest pain and dyspnea on hospital day 5, meropenem was replaced with vancomycin and dental treatment, including extraction of the right maxillary canine and remaining roots of first molar (Fig. 2a) and oral hygiene instructions were additionally provided.

Repeated blood culture resulted in no growth of any microorganisms after 5 days of incubation using the BacT/Alert 3D system (Sysmex-Biomerieux, Tokyo, Japan). To determine the causative pathogen, bronchoscopic BAL was performed and BAL fluid was collected. The BAL fluid was collected by the injection of normal saline into the right middle lobe (B5a), which contained the embolic lesion indicated by the CT imaging, followed by bronchial washing twice with normal saline injection to avoid contamination by commensal organisms. The BAL fluid sample was subcultured on Brucella HK (hemin, vitamin K1) RS (rabbit, sheep) blood agar (Kyokuto, Tokyo, Japan) and then incubated under an anaerobic condition at 35 °C. After 96 h of incubation, white colonies appeared on the agar plate. We identified the isolate as *Actinomyces species* (*Actinomyces spp.*) using the RapID ANA II systems (Amco, Tokyo, Japan). The minimum inhibitory concentrations of drugs determined by Dry Plate Eiken SB-03 (Eiken Chemical, Tokyo, Japan) showed that the isolate was highly susceptible to all β-lactams, clindamycin, and carbapenems (Table 1).

**Table 1:** Antimicrobial susceptibilities of the patient’s isolates

Antibiotics	MIC (μg/mL)
Penicillin G	≤0.25
Ampicillin	≤0.25
Piperacillin	≤8
Cefozopran	≤4
Cefmetazole	≤4
Latamoxef	≤4
Imipenem	≤1
Tosufloxacin	>4
Clindamycin	≤0.5
Minocycline	≤1
Chloramphenicol	≤4

*MIC* minimum inhibitory concentration

Two days after the start of vancomycin therapy and the completion of tooth extraction, the symptoms including fever and chest pain gradually improved. On hospital day 23, the patient was discharged and continued treatment with oral clindamycin. At the 1-month follow-up, since repeated CT showed almost complete disappearance of the lung lesions (Fig. 1b), antibiotic therapy was stopped. At the 3-month follow-up, there had been no recurrence of the symptoms and the lung lesions had completely resolved (Fig. 1b).

## Discussion

The present case demonstrates that i) *Actinomyces spp.* was the causative pathogen of the periodontal disease-associated SPE, and suggests that ii) appropriate anaerobic culture of BAL fluid would be useful for the identification of the pathogen of SPE and that iii) dental treatment is important for the initial management of SPE associated with periodontitis.

Although empirical therapy has often been performed for odontogenic infection because of the difficulty in identifying the causative pathogen, appropriate sample collecting and culturing technique may enable identification of the causative pathogen of odontogenic infection, including periodontal disease-associated SPE. Although previous reports have described that pathogenic bacteria could be identified in few cases of periodontal disease-associated SPE [1, 4], the present findings suggest that *Actinomyces spp.* can cause SPE and may not be a rare pathogen as the cause of periodontal disease-associated SPE, in which inappropriate sample culturing and collecting have often failed to identify the pathogen.

*Actinomyces spp*. are Gram-positive, predominantly anaerobic prokaryotic bacteria that reside in the oral cavity [5]. *Actinomyces spp*. have been reported as one of the causative bacteria of anaerobic lung abscesses [5]. However, *Actinomyces spp*. are fastidious bacteria and difficult to culture [5]. Thus, correct techniques for collecting samples and anaerobic culture are vital. *Actinomyces spp.* grow in an atmosphere of 6–10 % ambient CO_2_, and its colonies characteristically appear as “molar-tooth” or “bread-crumb” colonies after 3–7 days of anaerobic incubation [5]. For adequate growth, however, cultures should be observed for up to 21 days [5].

Using BAL fluid collection and the appropriate conditions for anaerobic incubation, we identified *Actinomyces spp.* as the causative pathogen of the SPE associated with the severe chronic periodontitis. The utility of the incubation of BAL fluid specimens has been reported for the diagnosis of anaerobic lung abscess [6]. Aggravated SPE develops into anaerobic abscess formation [7]. Thus, when SPE lesion develops into a microabscess or focal abscess, we can identify the responsible pathogen from BAL fluid collected from the abscessed lesion. In the present case, we obtained the BAL fluid from the lobe that contained the embolic lesion. Furthermore, oral commensal bacterial has been reported to rarely affect or contaminate BAL fluid specimens [8]. Thus, these findings strongly suggest that *Actinomyces spp.* identified in the BAL fluid was the causative pathogen of SPE in the present case.

For the culture of pathogenic *Actinomyces spp.,* the appropriate sample collecting technique is also important. The sample should be collected anaerobically with caution, since a BAL fluid culture of *Actinomyces spp.* may be falsely negative if the sample is exposed to air for more than 20 min [5]. Open lung biopsy has been reported to be also useful for obtaining uncontaminated samples for histological and microbiological conformation of SPE [9], but it is, of course, more invasive than BAL fluid collection.

The result of blood culture was negative at 5 days incubation with the automated blood culture system that is based on the detection of bacterial CO_2_ production by a colorimetric sensor [10]. In the usual protocol, blood cultures are incubated for 5–7 days; however, it is necessary to extend the incubation period for detecting microorganisms that proliferate slowly [11]. Similarly, Peretz et al. reported that slow growth microorganisms could be identified by subsequent Gram staining of the blood cultures that were identified as negative by an automated blood culture system at the end of the 7-day incubation [11]. Because *Actinomyces spp.* are fastidious bacteria that proliferate slowly, incubation of the blood culture for only 5 days might not be sufficient to identify causative *Actinomyces spp.,* resulting in a false-negative result in the present case.

We diagnosed the odontological condition of the present case as severe chronic periodontitis based on the oral findings [12], and regarded it as the infectious source of SPE. Chronic periodontitis usually shows a slow to moderate rate of progression. However, the complication of diabetes mellitus and heavy alcohol consumption has been related with the severity of periodontitis [13, 14]. Thus, in the present case, we supposed that the aggravation of chronic periodontitis was occurred in the immunocompromised setting associated with poorly controlled diabetes mellitus and heavy alcohol consumption, and that aggravated periodontitis subsequently caused SPE.

Dental treatment is important and sometimes even essential for the initial management of periodontitis-associated SPE. Although *Actinomyces spp.* are usually sensitive to most of the standard antibacterial agents [5] and were susceptible to carbapenems in the present case (Table 1), the symptoms of the patient were not at all improved by meropenem therapy alone. In contrast, the symptoms promptly improved after the mechanical dental treatment, suggesting that successful drainage of the infectious source by the dental treatment led to amelioration of the infection. Additionally, antibiotics are known to have limited effect on the bacteria embedded in biofilms that are formed in periodontal disease and dental caries if not combined with mechanical periodontal treatment or tooth extraction [15]. Thus we should consider dental treatment for the initial management of periodontitis-associated SPE in addition to appropriate antibiotics therapy.

We did not exclude the possibility that a shift in the antimicrobial regimen was potentially involved in the improvement of the symptoms in the present clinical course. Methicillin-resistant staphylococcus aureus (MRSA) has been reported to be a possible cause of SPE [4], and it is resistant to meropenem and susceptible to vancomycin. Thus, since the present case was unresponsive to the initial meropenem therapy, we changed the antibiotics to vancomycin before the culture results of BAL fluid were available. However, MRSA was not identified in any specimens, and the causative *Actinomyces spp*. are susceptible to both vancomycin and meropenem, which have favorable penetration in the pleural cavity.[16, 17] Therefore, we suppose that the shift in antimicrobial regimen did not very much contribute to the clinical improvement.

## Conclusion

The present case demonstrated that *Actinomyces spp.* can cause periodontal disease-associated SPE. Additionally, clinicians should consider collecting BAL fluid samples and performing appropriate anaerobic culture to identify the pathogen of SPE, and arranging for dental treatment as an initial management for periodontitis-associated SPE in addition to antibiotics therapy.

## Consent

Written informed consent was obtained from the patient for publication of this case report and any accompanying images. A copy of the written consent is available for review by the editor of this journal.
